# Comparative Evaluation of Supraclavicular Versus Infraclavicular Approach With Regard to Right Subclavian Vein Catheterisation by Blind Approach

**DOI:** 10.7759/cureus.93438

**Published:** 2025-09-28

**Authors:** Chintalapalli Ch Shanmuka Srikanth, Krishna Anusha Akula, Kamisetty Mani Bharath, Gattu Vijayalaxmi, Suresh Babu Sayana

**Affiliations:** 1 Department of Anaesthesiology, Konaseema Institute of Medical Sciences, Amalapuram, IND; 2 Department of Anaesthesiology, Mamata Medical College, Khammam, IND; 3 Department of Anaesthesiology, GSL Medical College, Rajahmundry, IND; 4 Department of Pharmacology, Government Medical College and General Hospital, Bhadradri Kothagudem, IND

**Keywords:** cardiac surgery, central venous catheterisation, infraclavicular approach, landmark-guided technique, randomised controlled trial, right subclavian vein, supraclavicular approach

## Abstract

Background

In settings where real-time ultrasonography (USG) is unavailable or delayed, landmark-guided central venous access remains essential. Anatomically, the right supraclavicular (SC) route offers a more direct course to the superior vena cava than the infraclavicular (IC) approach, which could shorten procedural time without increasing risk.

Methods

We conducted a prospective, single-centre, parallel-group randomised controlled trial in adult elective cardiac surgery patients (n=70; 35 per arm) comparing landmark-guided right subclavian venous catheterisation via SC versus IC approaches, using a standardised Seldinger technique. The primary outcome was total cannulation time. Secondary outcomes included access time, number of puncture attempts, observer-rated ease of guidewire/catheter passage, success rate and complications. Binary outcomes were analysed for all randomised patients; timing outcomes were assessed in procedures with complete records (n=62; SC: 32, IC: 30).

Results

SC significantly reduced procedure duration. The mean total cannulation time was 2.98±0.59 minutes (SC) versus 3.70±0.77 minutes (IC), with a mean difference of -0.72 minutes (95% confidence interval (CI): -1.07 to -0.37; p<0.001). The mean access time was 17.72±17.89 seconds (SC) versus 35.80±33.63 seconds (IC), with a mean difference of -18.1 seconds (95% CI: -32.0 to -4.2; p=0.012). The distribution of puncture attempts and observer-rated ease scores did not differ materially between groups. Overall success was high (94.3% SC versus 85.7% IC; Fisher’s exact test; p=0.428). Complications were infrequent: subclavian arterial puncture occurred in 2/35 (SC) versus 5/35 (IC) (p=0.428); no pneumothorax, haemothorax, haematoma or catheter malposition was observed.

Conclusions

In adult cardiac surgical patients, the SC approach achieves faster landmark-guided right subclavian venous access than the approach, without detectable compromises in success or safety. These findings support routine competence with the SC technique when ultrasound cannot be used and justify larger multicentre trials powered for rare complications and operator-learning effects.

## Introduction

The modern era of central venous access traces back to Werner Forssmann, who advanced a catheter from his arm vein into his right atrium in 1929; his pioneering work was later recognised with the Nobel Prize in 1956 [1]. Since then, central venous catheterisation has become integral to peri-operative and critical care practice for haemodynamic monitoring and therapy. The introduction of the Seldinger technique, which replaces the needle with a guidewire to place catheters, made large vein access simpler and safer. Monitoring central venous pressure (CVP) provides insight into right atrial pressure and, with clinical context, helps differentiate causes of circulatory instability and guide resuscitation [2].

Central venous access is obtained most commonly via the internal jugular, subclavian or femoral veins, for indications including haemodynamic monitoring, rapid volume resuscitation, vasoactive infusions, long-term chemotherapy, total parenteral nutrition, renal replacement therapy access, cardiopulmonary resuscitation (CPR) and difficult peripheral access [3]. Early clinical descriptions of jugular catheterisation appeared in the 1960s-1970s. The subclavian route remains favoured in many settings due to its large calibre, relative absence of valves, patient comfort and lower infection and thrombosis risk compared with femoral and often with internal jugular access [4].

The infraclavicular (IC) subclavian technique was first described by Aubaniac in 1952 and subsequently popularised for CVP monitoring by Wilson and colleagues in the early 1960s. However, the IC approach relies on landmarks that can be difficult to interpret and may be associated with complications such as arterial puncture and pleural injury. As an alternative, Yoffa introduced the supraclavicular (SC) approach in 1965, targeting the venous confluence near the clavisternomastoid angle. The right-sided SC route is usually preferred. It provides a short, almost linear course from the right subclavian/brachiocephalic vein to the superior vena cava, which facilitates guidewire and catheter passage [4], and it avoids the thoracic duct, which drains into the left venous angle, thereby reducing the risk of chylothorax [3]. It also maintains access during CPR and is less affected by body-habitus features that may reduce technical difficulty and certain complications [5,6].

Real-time ultrasonography (USG) improves cannulation by reducing attempts, increasing success, shortening procedure time and lowering complication rates. Nevertheless, USG may not always be immediately available or operable by trained personnel, especially when rapid access is required; therefore, landmark-guided (“blind”) techniques remain important skills. Contemporary reports suggest that the SC approach achieves high success with acceptable safety profiles across varied settings, including mechanically ventilated patients [7]. Comparative landmark-based studies have evaluated success, time to cannulation and complications, with several suggesting performance advantages for the SC route, although findings vary by population and method. Even under ultrasound guidance, differences in procedural dynamics between SC and IC access have been observed. In this context, access to the right subclavian vein using landmark-guided techniques remains clinically relevant. The present study focuses on a head-to-head comparison of SC versus IC approaches for right subclavian venous catheterisation in adult cardiac surgical patients, where timely, reliable access is essential [8,9].

The objective is to compare landmark-guided SC and IC approaches for right subclavian venous catheterisation. The primary outcome is total cannulation time. Secondary outcomes include access time, number of attempts (overall and among successful cases), ease of cannulation (guidewire and catheter), overall success rate and procedure-related complications (pneumothorax, haemothorax, arterial puncture, haematoma formation and catheter tip misplacement).

## Materials and methods

Study design and oversight

This was a prospective, randomised, parallel-group trial conducted in the Cardio-Thoracic and Vascular Surgery (CTVS) operating theatres of GSL Medical College and General Hospital between October 2018 and June 2020. The Institutional Ethics Committee approved the protocol (approval number: GSLMC/RC: S27-EC/S27-10/18). Written informed consent was obtained from all participants or their first-order relatives, as applicable. The trial compared two landmark-guided techniques for right subclavian venous catheterisation: the SC and IC approaches. The sample size targeted the detection of a ~1-minute between-group difference in total cannulation time, assuming a common standard deviation (SD) of ≈1.3 minutes, two-sided α of 0.05 and 90% power, yielding 35 patients per group.

Participants: Eligibility criteria

Adults of either sex, aged 20-65 years, scheduled for elective open-heart surgery were eligible. Exclusion criteria were as follows: refusal to participate, neck abnormalities (congenital/anatomical deformities, post-burn contracture, etc.), infection at the puncture site, deranged coagulation profile, known vascular abnormalities, contralateral pneumothorax and trauma to the clavicle/upper ribs or cervical spine. Pre-operative assessment included complete haemogram (haemoglobin (Hb%), total leukocyte count (TLC), differential leukocyte count (DLC), erythrocyte sedimentation rate (ESR) and platelet count), fasting and post-prandial blood glucose, serum urea and creatinine, electrolytes (Na⁺, K⁺ and Cl⁻), liver function tests, coagulation profile, prothrombin time (PT), activated partial thromboplastin time (APTT), international normalised ratio (INR), transthoracic echocardiography, chest radiograph posteroanterior (PA) view, 12-lead electrocardiogram (ECG) and additional investigations as clinically indicated.

Randomisation, allocation concealment and masking

Seventy consecutive eligible patients were randomised in a 1:1 ratio to SC or IC (n=35 per group) using a computer-generated sequence with variable block sizes. Allocation concealment was ensured with sequentially numbered, opaque, sealed envelopes opened in theatre immediately before cannulation. Blinding of operators and the in-theatre assessor was not feasible; patients were under general anaesthesia and therefore unaware of group assignment. To minimise assessment variability, the same senior cardiac anaesthetist performed all observer-rated ease assessments using a pre-approved departmental scale (see below).

Peri-operative care and positioning

Standard monitors (non-invasive blood pressure, pulse oximetry and continuous ECG) were applied. A peripheral intravenous line and a radial arterial cannula were placed. Anaesthesia was induced and maintained according to the unit’s standardised cardiac anaesthesia protocol. Patients were positioned supine with a slight Trendelenburg tilt; the head was rotated to the contralateral side, and a wedge was placed beneath the shoulder blades.

Interventions and procedural standardisation

All catheterisations employed a landmark-guided Seldinger technique. A 22-gauge (G) seeker needle was used to localise venous entry, followed by an 18G introducer needle, guidewire passage, tract dilatation and insertion of a triple-lumen central venous catheter, which was then secured and dressed. Correct placement was confirmed by aspiration of blood from all three catheter ports. Ultrasonography (USG) was reserved for rescue only. The right subclavian vein was used in all patients. A single primary operator, a cardiac anaesthetist with eight years’ independent experience in central venous catheterisation and a cumulative case volume of >300 landmark-guided subclavian lines (including >150 via the supraclavicular route), performed all first-line cannulations. If predefined failure criteria were met (see below), a senior anaesthetist with 12 years’ experience completed the procedure; this was recorded as failure per protocol.

SC approach

The skin entry point was 1 cm cephalad and 1 cm lateral to the junction of the lateral margin of the clavicular head of the sternocleidomastoid with the superior border of the clavicle (clavisternomastoid angle). After antisepsis and draping, the 22G seeker needle was introduced; once venous localisation was achieved, the 18G introducer was advanced. The bevel was initially oriented upwards and, after venous aspiration, turned downwards to discourage J-wire ascent into the internal jugular vein. Guidewire insertion, tract dilatation and catheter placement proceeded as standard.

IC approach

The puncture site was 1 cm inferior to the junction of the medial one-third and lateral two-thirds of the clavicle. After vein localisation with a 22G seeker needle, the 18G introducer was advanced with its bevel directed inferomedially to discourage J-tip guidewire (J-wire) passage into the contralateral subclavian or ipsilateral internal jugular vein. Subsequent steps mirrored the SC approach.

Outcomes and operational definitions

The primary outcome was total cannulation time, defined as the interval from insertion of the 22G seeker needle to confirmation of successful triple-lumen catheter placement by aspiration from all three ports (reported in decimal minutes).

Secondary outcomes were as follows: access time, which is the interval from 22G seeker insertion to aspiration of venous blood through the 18G introducer (seconds); number of attempts, which is the number of separate skin punctures with the 18G introducer to obtain venous blood (an attempt was defined as insertion of the 18G needle through the skin and its subsequent removal; maximum permitted attempts = 3); ease of cannulation, observer-rated on a departmental 5-point Likert-type scale scored separately for guidewire and catheter (1 = >2 attempts or failed procedure, 2 = second attempt with >1 manipulation, 3 = second attempt with no manipulation or first attempt with >1 manipulation, 4 = first attempt with one manipulation and 5 = first attempt with no manipulation) (the scale was developed by an intradepartmental panel and approved by the ethics committee); overall success rate; and complications, including pneumothorax, haemothorax, arterial puncture, haematoma and catheter-tip misplacement/displacement.

Failure criteria were as follows: >3 attempts without successful venous aspiration, need to change site or catheter size due to inability to pass the catheter into the right subclavian vein or operator change. In failures, right internal jugular vein catheterisation was performed; real-time USG rescue was available if required.

Complication Ascertainment

After thoracotomy, surgeons specifically inspected for pneumothorax, haemothorax and haematoma; clinical surveillance continued post-operatively according to institutional practice.

Analysis populations and handling of missing data

All randomised patients (n=70) were included for analyses of success/failure and complications (intention-to-treat for binary outcomes). Timing outcomes (access time and total cannulation time) were analysed in procedures with complete timing records (n=62: SC, n=32; IC, n=30) using a complete-case approach; no imputation was performed.

Statistical analysis

Continuous variables were summarised as mean (SD) or median and interquartile range (IQR), as appropriate. Distributional assumptions were assessed (Shapiro-Wilk and visual inspection). Between-group comparisons used two-sample t-tests (Welch’s unequal-variance t-test when variances differed; applied to the access time comparison) for approximately normally distributed data and Mann-Whitney U for non-normal data (prespecified for the ordinal ease scores). Categorical variables were compared using Pearson’s χ² test; when any expected cell count was <5, Fisher’s exact test was used. Two-sided p<0.05 denoted statistical significance. Where applicable, 95% confidence intervals (CIs) were calculated. Inference was confirmatory only for the single prespecified primary outcome (two-sided α=0.05). Secondary outcomes were analysed as exploratory; p-values are nominal and are presented with effect sizes and 95% confidence intervals, and are interpreted cautiously rather than used for formal claims. Accordingly, no multiplicity adjustment was planned.

## Results

Participant flow and baseline characteristics

Seventy patients were randomised (n=35 per group) to right subclavian venous catheterisation via the SC or IC landmark approach. All 70 participants were included in baseline comparisons and binary outcomes (success and complications). Timing outcomes were analysed in procedures with complete time records (n=62: SC, n=32; IC, n=30); one successful SC cannulation lacked a timing entry (Table 1).

**Table 1:** Baseline characteristics SC: supraclavicular, IC: infraclavicular, SD: standard deviation

Characteristic	Group 1 (SC) (n=35)	Group 2 (IC) (n=35)	p-value (test)
Age, years (mean±SD)	32.91±10.31	37.25±11.22	0.100 (Student’s t-test)
Height, cm (mean±SD)	159.65±8.26	160.02±7.32	0.843 (Student’s t-test)
Weight, kg (mean±SD)	56.51±7.00	57.28±5.17	0.602 (Student’s t-test)
Sex, number (%) (female)	18 (51.4%)	19 (54.3%)	0.811 (Pearson χ² test)
Sex, number (%) (male)	17 (48.6%)	16 (45.7%)	

Procedural performance

Compared with the IC approach, the SC approach achieved venous access more rapidly and with fewer slow outliers. Among procedures with complete timing (n=62), 93.8% of SC cannulations were completed within ≤30 seconds versus 63.3% for IC, while IC contributed nearly all events beyond 60 seconds (31-60 seconds, 26.7%; 91-120 seconds, 6.7%; and 121-160 seconds, 3.3%). The mean difference of -18.1 seconds (95% CI: -32.0 to -4.2; Welch t-test, p=0.012) indicates a clear timing advantage for SC, consistent with the visibly tighter distribution in the ≤30 second band (Table 2).

**Table 2:** Access time (seconds)* Access time was defined as the interval from the first insertion of the 22G seeker to the first confirmed venous aspiration with the 18G introducer. Analysed only in procedures with complete timing logs (n=62: SC, 32; IC, 30). Values are numbers (%) by 30-second bands and mean±SD. Between-group mean difference: -18.1 seconds (95% CI: -32.0 to -4.2), favouring SC; Welch unequal-variance t-test, p=0.012. *Analysed among procedures with complete timing SC: supraclavicular, IC: infraclavicular, SD: standard deviation, CI: confidence interval

Category (seconds)	Group 1 (SC) (n=32)	Group 2 (IC) (n=30)
1-30	30 (93.8%)	19 (63.3%)
31-60	0 (0%)	8 (26.7%)
61-90	2 (6.3%)	0 (0%)
91-120	0 (0%)	2 (6.7%)
121-160	0 (0%)	1 (3.3%)
Mean±SD (seconds)	17.72±17.89	35.80±33.63

Total cannulation time

Total cannulation time favoured SC, with the bulk of SC procedures falling in the 2.1-4.0 minute band (93.8%) and virtually no cases exceeding 6 minutes. By contrast, IC showed a broader spread: 70% in 2.1-4.0 minutes, 26.7% in 4.1-6.0 minutes and 3.3% in >6 minutes. The mean difference of -0.72 minutes (≈43 seconds; 95% CI: -1.07 to -0.37; p<0.001) demonstrates both a shorter average procedure and fewer prolonged cannulations with SC (Table 3).

**Table 3:** Total cannulation time (minutes, decimal format)* Total cannulation time was defined as the interval from 22G seeker insertion to successful aspiration from all three catheter ports after placement. Analysed only in procedures with complete timing logs (n=62: SC, 32; IC, 30). Values are numbers (%) by minute bands (decimal minutes) and mean±SD. Between-group mean difference: -0.72 minutes (95% CI: -1.07 to -0.37), favouring SC; Student’s t-test, p<0.001. *Analysed among procedures with complete timing SC: supraclavicular, IC: infraclavicular, SD: standard deviation

Category (minutes)	Group 1 (SC) (n=32)	Group 2 (IC) (n=30)
1.0-2.0	1 (3.1%)	0 (0%)
2.1-4.0	30 (93.8%)	21 (70%)
4.1-6.0	1 (3.1%)	8 (26.7%)
>6.0	0 (0%)	1 (3.3%)
Mean±SD (minutes)	2.98±0.59	3.70±0.77
p-value	<0.001 (Student t)	

Ease of cannulation (ordinal scale)

Ease-of-passage scores were high in both groups, with most procedures rated “5” (very easy) for both guidewire (85.7% (SC) versus 71.4% (IC)) and catheter (82.9% (SC) versus 71.4% (IC)). Although the proportions numerically favoured SC, ordinal comparisons were not statistically significant (guidewire, p=0.166; catheter, p=0.266). Only scores 1, 3 and 5 occurred, suggesting a ceiling effect whereby both techniques generally allowed effortless hardware advancement in this cohort (Table 4).

**Table 4:** Ease of cannulation (guidewire and catheter) (number (%)) Observer-rated ease of passage on a departmental 5-point ordinal scale (1 = very difficult/resistance, 3 = moderate and 5 = very easy), recorded separately for guidewire advancement and catheter threading at the time of the attempt. Only scores 1, 3 and 5 occurred (no observations at 2 or 4). Data are numbers (%). Between-group comparison: Mann-Whitney U, p=0.166 (guidewire) and p=0.266 (catheter). SC: supraclavicular, IC: infraclavicular

Scale value	Group 1 (SC)	Group 2 (IC)	Total
Guidewire			
1	3 (8.6%)	5 (14.3%)	8 (11.4%)
3	2 (5.7%)	5 (14.3%)	7 (10%)
5	30 (85.7%)	25 (71.4%)	55 (78.6%)
p-value (Mann-Whitney U)	0.166		
Catheter			
1	3 (8.6%)	5 (14.3%)	8 (11.4%)
3	3 (8.6%)	5 (14.3%)	8 (11.4%)
5	29 (82.9%)	25 (71.4%)	54 (77.1%)
p-value (Mann-Whitney U)	0.266		

Number of puncture attempts (18G)

First-pass success was common with both approaches (82.9% (SC) versus 71.4% (IC)), and the share requiring ≥2 attempts was similar (17.1% (SC) versus 28.6% (IC)). The distribution across 1/2/3 attempts did not differ (χ², p=0.523), and a sensitivity comparison of 1 versus ≥2 attempts likewise showed no material difference (Fisher’s exact test, p=0.394). These data indicate comparable needle-redirection burdens between SC and IC in routine practice (Table 5).

**Table 5:** Number of 18G puncture attempts (number (%)) Number of 18G venepuncture attempts per patient with the assigned approach; includes all randomised procedures (SC, 35; IC, 35). Data are numbers (%). Primary comparison: Pearson χ² across 1/2/3 attempts (p=0.523). Sensitivity (prespecified): 1 versus ≥2 attempts, Fisher’s exact test, p=0.394, confirming no material difference. SC: supraclavicular, IC: infraclavicular

Attempts	Group 1 (SC) (n=35)	Group 2 (IC) (n=35)	Total	p-value (Pearson χ² test)
1	29 (82.9%)	25 (71.4%)	54 (77.1%)	0.523
2	3 (8.6%)	5 (14.3%)	8 (11.4%)	
3	3 (8.6%)	5 (14.3%)	8 (11.4%)	

Success and safety outcomes

Overall success rates were high (94.3% (33/35) (SC) versus 85.7% (30/35) (IC)); the absolute difference of 8.6 percentage points did not reach statistical significance (Fisher’s exact test, p=0.428). Thus, within this sample, both landmark routes reliably achieved right subclavian venous access, with no demonstrable difference in success probability (Table 6).

**Table 6:** Overall success (number (%)) Overall procedural success was defined as completion of right subclavian venous catheterisation via the allocated approach without conversion. Failures denote the inability to complete cannulation by the allocated route. Data are numbers (%). Between-group comparison: Fisher’s exact test, p=0.428. SC: supraclavicular, IC: infraclavicular

Outcome	Group 1 (SC) (n=35)	Group 2 (IC) (n=35)	p-value (Fisher’s exact test)
Successful	33 (94.3%)	30 (85.7%)	0.428
Failed	2 (5.7%)	5 (14.3%)	

Complications

Complications were uncommon. Accidental subclavian arterial puncture occurred in 2/35 (5.7%) in SC versus 5/35 (14.3%) in IC cases (p=0.428), while no pneumothorax, haemothorax, haematoma or catheter malposition was detected intra-operatively or in the immediate post-operative period in either group. Within the limits of the sample size, these results suggest a low complication burden for both approaches (Table 7).

**Table 7:** Complications (number (%)) Periprocedural complications recorded intra-operatively and in the immediate post-operative period. Accidental subclavian arterial puncture was uncommon, with no significant between-group difference (Fisher’s exact test, p=0.428). No cases of pneumothorax, haemothorax, haematoma or catheter malposition were observed. Values are numbers (%). SC: supraclavicular, IC: infraclavicular

Complication	Group 1 (SC) (n=35)	Group 2 (IC) (n=35)	p-value
Subclavian arterial puncture	2 (5.7%)	5 (14.3%)	0.428 (Fisher’s exact test)
Pneumothorax	0	0	-
Haemothorax	0	0	-
Haematoma	0	0	-
Catheter malposition	0	0	-

Summary of primary findings

Taken together, the timing metrics consistently favour the supraclavicular route, showing both faster central venous access and shorter total cannulation times with fewer slow outliers, while puncture attempts, ease ratings, overall success rates and observed complications were similar between approaches within this cohort.

## Discussion

Principal findings

In this randomised comparison of landmark-guided right subclavian venous catheterisation, the SC approach achieved materially faster performance than the IC approach for both access time (mean difference: -18.1 seconds, 95% confidence interval (CI): -32.0 to -4.2) and total cannulation time (mean difference: -0.72 minutes, 95% CI: -1.07 to -0.37). Procedural facility (ordinal ease scores), the distribution of puncture attempts and overall success rates were similar between groups. Complications were infrequent, with no pleural events or malposition detected and a non-significant difference in subclavian arterial puncture. These results indicate that, when ultrasound is unavailable or delayed, the SC route can expedite reliable central access without apparent trade-offs in success or safety in adult cardiac surgical patients.

Interpretation and mechanistic context

The time advantage with SC access is anatomically plausible. The right SC trajectory targets the venous confluence at the clavisternomastoid angle and offers a straighter, shorter path to the superior vena cava than the IC route, reducing angular turns and the need for manipulation during guidewire and catheter passage [4,10,11]. In our cohort, this translated into a pronounced left shift in the access time distribution; most SC cannulations achieved venous entry within 30 seconds, while maintaining similar first-pass facility and attempts. The use of a seeker needle, deliberate bevel orientation to discourage J-wire ascent into the internal jugular vein and uniform right-sided cannulation likely further minimised frictional steps in the SC arm.

Comparison with prior evidence

Our findings align with landmark-guided trials that reported shorter cannulation metrics with SC relative to IC access and are consistent with observational series showing high SC success across varied settings, including mechanically ventilated patients. Even in ultrasound-guided head-to-head work, procedural segments (visualisation, puncture and catheterisation) have been shorter for SC despite comparable success, indicating an anatomical efficiency that persists regardless of imaging. Conversely, earlier comparisons that emphasised IC familiarity did not consistently show speed advantages, likely reflecting operator preference and learning curve effects; our single-operator design reduces that source of variability and clarifies the signal in favour of SC when techniques are standardised [12-14].

Procedural facility, success and learning considerations

Despite faster times with SC, ordinal ease scores and puncture attempts were indistinguishable between groups, suggesting that once landmarks and needle orientation are mastered, both approaches can be executed with similar subjective and objective facility. The overall success rate was high in both arms (94.3% (SC) versus 85.7% (IC)) without statistical separation (Fisher’s exact test, p=0.428), which is consistent with a trial powered for time, not for modest differences in success proportions. Taken together, these observations support a pragmatic message: the efficiency gain with SC does not come at a cost to first-pass performance or completion. Examined as prespecified subscales, guidewire and catheter ease scores each showed no material between-group difference (Mann-Whitney U test, p=0.166 and p=0.266, respectively), reinforcing that the SC time gain was not accompanied by greater subjective difficulty [14,15].

Safety profile

No pneumothorax, haemothorax, haematoma or catheter malposition occurred, and arterial puncture was infrequent with no significant between-group difference (2/35 (SC) versus 5/35 (IC); Fisher’s exact test, p=0.428). The low event burden likely reflects rigorous technique, seeker needle use, uniform right-sided access and continuous intra-operative surveillance with direct thoracic inspection after sternotomy. Prior reports have suggested fewer pleural complications with SC, attributed to a higher target relative to the lung apex. While our data are directionally reassuring, they should be interpreted with caution, given the limited power for rare events [16,17].

Strengths and limitations

Key strengths include prospective randomisation with allocation concealment, a single primary operator (reducing inter-operator variability), a standardised protocol for both techniques and a consistent assessor using a prespecified ordinal scale. Timing denominators were explicit (complete case analysis in 62/70 procedures), and missingness was minimal and unlikely to bias group comparisons. Although we prespecified reporting puncture attempts among successful cases, these data were not separately tabulated; future studies should include this stratification to more cleanly attribute time gains to first-pass performance.

Limitations merit emphasis. First, this was a single-centre study in adult cardiac surgical patients, which may limit external validity to other populations and provider mixes. Second, the trial was powered for a one-minute difference in total cannulation time proportions, and infrequent complications were not the primary targets and remain underpowered. Third, masking of operators and the in-theatre assessor was not feasible; although a uniform assessor mitigates variability, subjective ease ratings could still be influenced by contextual cues. Finally, we used landmark guidance exclusively (ultrasound reserved for rescue); results therefore inform settings where ultrasound is unavailable, not a head-to-head of landmark versus real-time imaging. Single-operator conduct by an experienced cardiac anaesthetist reduces inter-operator variability but may limit generalisability to less-experienced providers.

Clinical implications

For time-critical central access in theatres and critical care, particularly when ultrasound is unavailable, impractical or delayed, the SC approach offers a practical, anatomy-supported advantage in speed while maintaining high success and a low complication burden in experienced hands. Training curricula should preserve proficiency in both landmark techniques but place deliberate emphasis on SC surface anatomy (clavisternomastoid angle), bevel/wire strategies to prevent cephalad misdirection and the routine use of a seeker needle to minimise vascular or pleural injury.

Future directions

Two avenues warrant priority. First, multicentre pragmatic trials with diverse operators and case mixes should evaluate SC versus IC under landmark guidance, powered for safety endpoints and including learning curve analyses. Second, comparative effectiveness studies should test frontline ultrasound versus landmark SC in realistic workflow constraints (e.g., emergency access and resource-limited environments), integrating operator time, needling time, total cannulation time and patient-centred outcomes. Embedding cost and throughput measures would further support adoption decisions.

Within the constraints of landmark guidance, the right SC route provides faster venous access than the IC route without sacrificing success or safety in this cardiac surgical cohort. These data, together with anatomical logic and convergent external evidence, support routine competence with the SC approach and justify larger, methodologically robust studies to refine technique selection across settings.

## Conclusions

In this randomised comparison of landmark-guided right subclavian venous catheterisation, the SC approach delivered significantly faster access (17.72±17.89 seconds versus 35.80±33.63 seconds; p=0.012; mean difference: -18.1 seconds; 95% CI: -32.0 to -4.2) and shorter total cannulation time (2.98±0.59 minutes versus 3.70±0.77 minutes; p<0.001; mean difference: -0.72 minutes; 95% CI: -1.07 to -0.37) than the IC approach, without compromises in procedural facility, overall success (94.3% versus 85.7%; Fisher’s exact test, p=0.428) or safety (arterial puncture: 5.7% versus 14.3%; Fisher’s exact test, p=0.428; no pneumothorax, haemothorax, haematoma or malposition).

When ultrasound is unavailable or delayed, the SC route offers a practical efficiency advantage while maintaining a low complication burden in experienced hands. Training programmes should therefore emphasise SC surface anatomy, bevel/wire orientation and the routine use of a seeker needle. Larger multicentre studies, powered for infrequent adverse events and incorporating operator learning curves and real-world workflow (including comparisons with frontline ultrasound), are warranted to refine generalisability and guide technique selection across settings.
